# Subcortical Hypermetabolism Associated With Cortical Hypometabolism Is a Common Metabolic Pattern in Patients With Anti-Leucine-Rich Glioma-Inactivated 1 Antibody Encephalitis

**DOI:** 10.3389/fimmu.2021.672846

**Published:** 2021-09-20

**Authors:** Xiaobin Zhao, Shaokun Zhao, Yaojing Chen, Zhanjun Zhang, Xiaotong Li, Xiao Liu, Ruijuan Lv, Qun Wang, Lin Ai

**Affiliations:** ^1^Department of Nuclear Medicine, Beijing Tiantan Hospital, Capital Medical University, Beijing, China; ^2^State Key Laboratory of Cognitive Neuroscience and Learning & International Data Group/McGovern Institute for Brain Research, Beijing Normal University, Beijing, China; ^3^Beijing Aging Brain Rejuvenation Initiative Centre, Beijing Normal University, Beijing, China; ^4^Department of Neurology, Beijing Tiantan Hospital, Capital Medical University, Beijing, China

**Keywords:** fluorodeoxyglucose, metabolic pattern, positron emission tomography, LGI1, autoimmune encephalitis

## Abstract

**Purpose:**

Brain 18F-fluorodeoxyglucose positron emission tomography (FDG PET) is a sensitive technique for assisting in the diagnosis of patients with anti-leucine-rich glioma-inactivated 1 (LGI1) antibody encephalitis. However, the common pattern of this disorder assessed by FDG PET remains unknown. The present study aimed to explore the glucose metabolic patterns of this disorder based on PET voxel analysis.

**Methods:**

This retrospective study enrolled 25 patients with anti-LGI1 encephalitis, who were admitted in Beijing Tiantan Hospital between September 2014 and July 2019. The glucose metabolic pattern was compared between the included patients and 44 age- and gender-matched healthy controls using Statistical Parametric Mapping. Then, the correlation between the metabolic pattern and scaled activities of daily living (ADLs) of the patients was assessed.

**Results:**

The median time from symptom onset to PET scans was 9 w (range:2-53w). The groupwise analysis revealed that patients with anti-LGI1 encephalitis had left hippocampal hypermetabolism and hypometabolism in almost all neocortical regions. The individual-level results showed most patients presented a decreased metabolism in neocortical regions, as well as an increase in metabolism in the hippocampus and basal ganglia. Furthermore, the metabolic gradient between hippocampus and neocortical regions was positively associated with the ADLs (frontal lobe, r=0.529, P=0.008; parietal lobe, r=0.474, P=0.019; occipital lobe, r=0.413, P=0.045; temporal lobe, r=0.490, P=0.015), respectively. In addition, the patients with facio-brachial dystonic seizures (FBDS) presented bilateral putamen hypermetabolism, when compared to patients without FBDS and healthy controls.

**Conclusion:**

Subcortical hypermetabolism associated with cortical hypometabolism presented with a common metabolic pattern in patients with anti-LGI1 encephalitis in the present study. The resolution of the metabolic gradient of the hippocampal hypermetabolism and neocortical hypometabolism may bring about improved clinical neurologic disability.

## Introduction

Anti-leucine-rich glioma-inactivated 1 (LGI1) encephalitis is the most common limbic encephalitis with antibodies targeting neuronal surface antigens (1). Patients with anti-LGI1 encephalitis present with progressive memory alteration, psychiatric manifestation and seizures, suggesting the involvement of the limbic system. The most characteristic seizures are facio-brachial dystonic seizures (FBDS), which are characteristic of anti-LGI1 encephalitis, and these are observed in approximately half of the patients (2, 3).

Early diagnosis means the early immunosuppressive treatment, which is associated with subsequent improved cognitive impairment and preventive irreversible lesions, including hippocampal atrophy (4, 5). At present, the initial diagnosis of anti-LGI1 encephalitis is mainly based on the clinical manifestations, MRI, and antibody testing (1). Nonetheless, the clinical overlaps of various autoimmune encephalitis (AE) subtypes impede the differential diagnosis (6, 7). Furthermore, the brain MRI findings of T2WI/FLAIR hyperintensities, which involve the medial temporal lobe, are often absent at disease onset (8, 9), specifically at the FBDS stage. In addition, the prolonged time taken for results to return and inaccessibility in many institutions limit the clinical application of the antibodies testing.

Given these aforementioned AE descriptions, 18F-FDG PET can be a potential biomarker for AE diagnosis (1, 10–12). The higher uptake on 18F-FDG PET in otherwise normal mesial temporal lobe structures shown on the MRI indicates that using 18F-FDG PET can be more sensitive than MRI in assisting the diagnosis (10, 13). Furthermore, 18F-FDG PET can be more sensitive to abnormalities in patients with AE, when compared to assessments using electroencephalogram (EEG) and cerebrospinal fluid (CSF) inflammatory markers (8).

Most previous 18F-FDG PET studies on anti-LGI encephalitis have been limited to visual inspection with an inferior sensitivity to voxel analysis (9, 14–16), or the use of a relatively smaller sample size with heterogenous results for metabolic pattern analysis (17–20). The present study aimed to determine the brain common patterns of glucose metabolism changes in a relatively large sample of patients with anti-LGI1 encephalitis using Statistical Parametric Mapping (SPM) analysis. Furthermore, the present study aimed to determine the correlation of the metabolism pattern with the daily activities of anti-LGI1 encephalitis patients.

## Methods

### Patients

The present retrospective study was approved by the Ethics Committee of the Beijing Tiantan Hospital, Capital Medical University, and each participant provided a written informed consent. Using the database of the PET center, the investigators identified patients with anti-LGI1 encephalitis, who were admitted in Beijing Tiantan Hospital and underwent 18F-FDG PET between September 2014 and July 2019. These patients were cross-referenced with the Tiantan Hospital Neurology Department database. Part of these patients were previously reported (21). The inclusion criteria were, as follows: (1) patients who were admitted in the Neurology Department; (2) the clinical characteristics of the patient was detailed and complete; (3) patients with a relatively acute or subacute disease course. Two neurological experts (L.R. and W.Q.) performed the diagnoses of anti-LGI1 encephalitis based on the consensus criteria (1).

### Clinical Evaluation

The activities of daily living (ADL) were gained by adding the Katz activities of daily living scale (22) to the Lawton instrumental activities of daily living scale (23). For male patients, 11 was set as normal, 10-7 was set as mild damage, 6-3 was set as moderate damage, and 2-0 was set as severe damage. For female patients, 14 was considered as normal, 13-9 was considered as mild damage, 8-4 was considered as moderate damage, and 3-0 was considered as severe damage. Furthermore, scaled ADL (ADLs) was produced by defining normal as 0, mild damage as 1, moderate damage as 2, and severe damage as 3.

### LGI1 Antibody Detection

For all patients, the serum and CSF antibody were detected, including NMDA receptor, LGI1, and contactin-associated protein-2, α-amino-3-hydroxy-5-methyl-4-isoxazolepropionic acid receptor, and γ-aminobutyric acid type B. The serum and CSF samples were tested for the presence of LGI1 antibodies using both commercially fixed cell-based assays (CBAs) in biochip form (Euroimmun, Lübeck, Germany), and immunohistochemical analyses in the Neuroimmunology Laboratory of Peking Union Medical College Hospital. The stained biochips were investigated under a fluorescence microscope (Eurostar 3 Plus, Euroimmun, Germany). The decision if antibody was present in the tested samples was made by two experienced investigators using the signal of the surrounding fields as negative controls.

### The 18F-FDG PET Protocol and Imaging Analysis

At the time of diagnosis, all participants underwent brain study. None of these patients received sedative drugs before the PET imaging. After fasting for 4-6 hours, 18F-FDG was intravenously injected at a dose of 3.7-5.0 MBq/kg. The pre-injection blood glucose levels were confirmed to be ≤8 mmol/L. The participants were isolated in a dedicated room during the uptake period. PET acquisitions were performed at 45–60 and 60–90 post-injection minutes for the brain and whole-body examinations, respectively. A brain PET scan was performed in the 3D-time-of-flight mode for 10 minutes. A whole-body (including brain region) 18F-FDG PET scan was performed for approximately 20-30 minutes. The brain imaging data were reconstructed using ordered subset expectation maximization methods, with four iterations and eight subsets, as well as smoothing with a 5-mm full-width at half-maximum filter.

The GE Advanced Workstation 4.6 software package (GE Healthcare) was used to generate the 18F-FDG PET images, and these were visually examined by two board-certified nuclear medicine physicians (Q.Z. and A.l.) with vast experience in reading PET/CT results (>10 years). These reviewers carefully checked the PET images for abnormalities in the hippocampus, basal ganglia, and neocortex. Furthermore, they also assessed the abnormality lateralization in case these were present. All reviewers were blinded from the clinical diagnosis of the patients or controls. Contradictions in the imaging evaluation were resolved through discussion, until a consensus was reached.

### MRI Protocol and Imaging Review

Clinical MRIs were performed according to the institutional protocol using a 3-T scanner (Genesis Signa and Signa HDe) and Siemens (Trio Tim and Verio). The brain MRI scans were evaluated by two neuroradiologists (C.Q. and W.K.), who were blinded to the patient’s diagnosis. In the present study, the T1WI and T2WI/FLAIR signals, and some of the pre- and post-gadolinium-enhanced T1WI sequences were reviewed and rated as consistent or inconsistent with AE. The differences in rating between the two reviewers were reconciled by discussion.

### Procedure for the PET Data Pre-Processing

The raw 18F-FDG PET images were pre-processed using SPM software version 12 (Wellcome Trust Centre for Neuroimaging, London, UK; https://www.fil. ion.ucl.ac.uk/spm/software/spm12/, default parameter settings). Initially, the raw data was transformed to NIfTI data, followed by realignment. Then, the brain images were spatially normalized into the stereotactic standard space based on the Montreal Neurological Institute (MNI) template using the ‘Old Normalize Tool’ of the SPM12. Stereotactically normalized images were smoothed using an isotropic 3D Gaussian kernel of 8-mm full-width half-maximal for the voxel-based analysis. Furthermore, the original normalized images underwent region of interesting (ROI) analysis. The voxel intensities in both smoothed and original normalized images were proportionally scaled to the average voxel intensity over the cerebellum. Furthermore, the investigators obtained the ratio of the standardized uptake value (SUVr). The cerebellum was used as the reference brain region, since the autoimmune encephalitis is less likely to affect this.

### Voxel-Based Statistical Testing

Regarding the group analysis, the investigators performed voxel-by-voxel between-group comparisons of the scaled 18F-FDG images. The statistical significance was set at P=0.001 (Family-Wise Error [FWE] = 0.001 for multiple comparisons and effects in clusters [KE]> 30) for comparisons between patients with anti-LGi1 encephalitis and the control group, between patients with FBDS and the control group, and between patients with PET scans < 12 weeks from symptom onset and those with PET scan ≥ 12 weeks from symptom onset, respectively. In addition, the statistical significance was set at P=0.001 without multiple comparison correction (effects in clusters [KE] >30) for comparison between patients with FBDS and those without FBDS, due to the small sample size. For the two-sample t-test in the SPM procedure, the investigators used a control group that comprised of 44 normal participants without a history of neurological or psychiatric disorders (age: 49.6 ± 18.9 years old, 14 women; there was no significant difference between all patients group and control group, in terms of age and gender, P=0.068 and P=0.291, respectively). The SPM analysis results were confirmed through visual assessment. For the individual analysis, the scaled 18F-FDG brain scans underwent voxel-by-voxel comparisons with the aforementioned control group (one-sided two-sample t-tests for hypo- and hyper-metabolism; P=0.001, uncorrected for multiple comparisons, no non-sphericity correction; KE >30).

### Statistical Analysis

In order to analyze the relationship between metabolic abnormalities in specific brain regions with ADL, the investigators chose the following ROIs: bilateral frontal lobe, bilateral hippocampus, and bilateral putamen. These ROIs were summarized from relatively small ROIs of Anatomical Automatic Labeling (AAL) templates (**Supplemental Table 1**). These ROIs were defined based on the individual- and group-level results obtained from the SPM analysis, as well as according to the visually recognized region identified by the nuclear medicine physician. The investigators calculated the mean SUVr in each ROI using the WFU Pick Atlas software and AAL-based masking. The association between the SUVr ratio of the hippocampus to neocortical regions and ADLs was determined using Spearman test, since the ratio data was non-normally distributed.

## Results

### Clinical Characteristics of Patients

Among the 28 patients who met the criteria for anti-LGI1, 25 patients (20 men, five women; median age: 60 years old) were included in the final analysis. Three patients were excluded due to poor image quality. **Table 1** summarizes the clinical characteristics of the included patients. The pre-scan symptom duration was a median of nine weeks (interquartile ranges [IQR]: 13; range:2-52). The duration between the MRI and 18F-FDG PET scans was a median of five days (IQR: 9; range:0-26). A total of 21 (84%) patients underwent a whole-body PET scan, and none of the results revealed a tumor. Furthermore, nine (36%) patients presented with FBDS. Moreover, 14 (56%) and eight (32%) patients presented with psychiatric symptom and memory impairment, respectively. The median ADLs at the time of PET scanning was 2 (interquartile range: 1.5). Furthermore, eight (32%) patients were treated with steroids within 24 hours before the PET scan. All 25 (100%) patients tested positive for LGI1 antibodies, in both the serum and CSF (92%), with two (8%) patients showing only in the CSF or serum. The patients tested negative for other antibodies of interest.

**Table 1:** Clinical characteristics of the patients included in the study.

Patient no.	Antibody positive findings in Serum or CSF	Age, y	Sex	Duration of symptoms before presentation (wk)	The duration of symptom onset and 18F-FDG PET (wk)	The duration between MRI and 18F-FDG PET (d)	ADLs at the time of 18F-FDG PET	FBDS	Memory impairment	Psychiatric symptom	Treated with steroids within 24h before 18F-FDGPET	Whole body scan or brain scanning
1	Both	59	M	2	3	3	3	N	N	N	Y	W
2	Both	70	M	26	28	15	2	N	Y	Y	Y	W
3	Both	34	F	2	3	11	2	N	N	Y	Y	W
4	Both	67	F	1	2	2	3	N	Y	Y	Y	W
5	Both	63	M	18	19	4	2	Y	Y	Y	N	W
6	Both	56	M	52	53	0	2	N	N	Y	N	W
7	Both	61	M	9	10	7	2	N	Y	Y	N	B
8	Both	41	F	4	5	4	2	N	N	N	N	W
9	Serum	15	M	1	2	3	1	N	N	N	N	W
10	Both	58	M	2	4	2	1	Y	Y	N	N	W
11	Both	49	M	13	13	3	1	N	N	Y	N	B
12	CSF	61	M	13	14	25	2	Y	N	N	N	W
13	Both	34	M	6	8	5	1	N	Y	Y	Y	W
14	Both	64	M	8	9	2	1	Y	N	N	N	W
15	CSF	65	M	39	40	16	2	N	N	N	Y	W
16	Both	60	M	13	14	7	3	N	N	Y	N	W
17	Both	78	M	0	2	8	3	Y	N	Y	N	W
18	Both	68	F	52	53	13	1	N	Y	Y	Y	W
19	Both	60	M	6	6	4	1	Y	N	N	N	W
20	Serum	39	F	1	2	5	1	N	N	N	N	B
21	Both	54	M	7	7	26	1	Y	Y	Y	N	W
22	Both	66	M	4	5	11	2	Y	N	N	N	W
23	Both	59	M	13	13	22	2	Y	N	Y	N	B
24	Both	67	M	17	19	3	3	N	N	Y	Y	W
25	Both	47	M	9	9	3	1	N	N	N	N	W

F, female; M, male; ADLs, activities daily life scale; FBDS, faciobrachial dystonic seizure; Y, yes; N, no; B, brain; W, whole body.

### Visual Inspection

**Figure 1:**
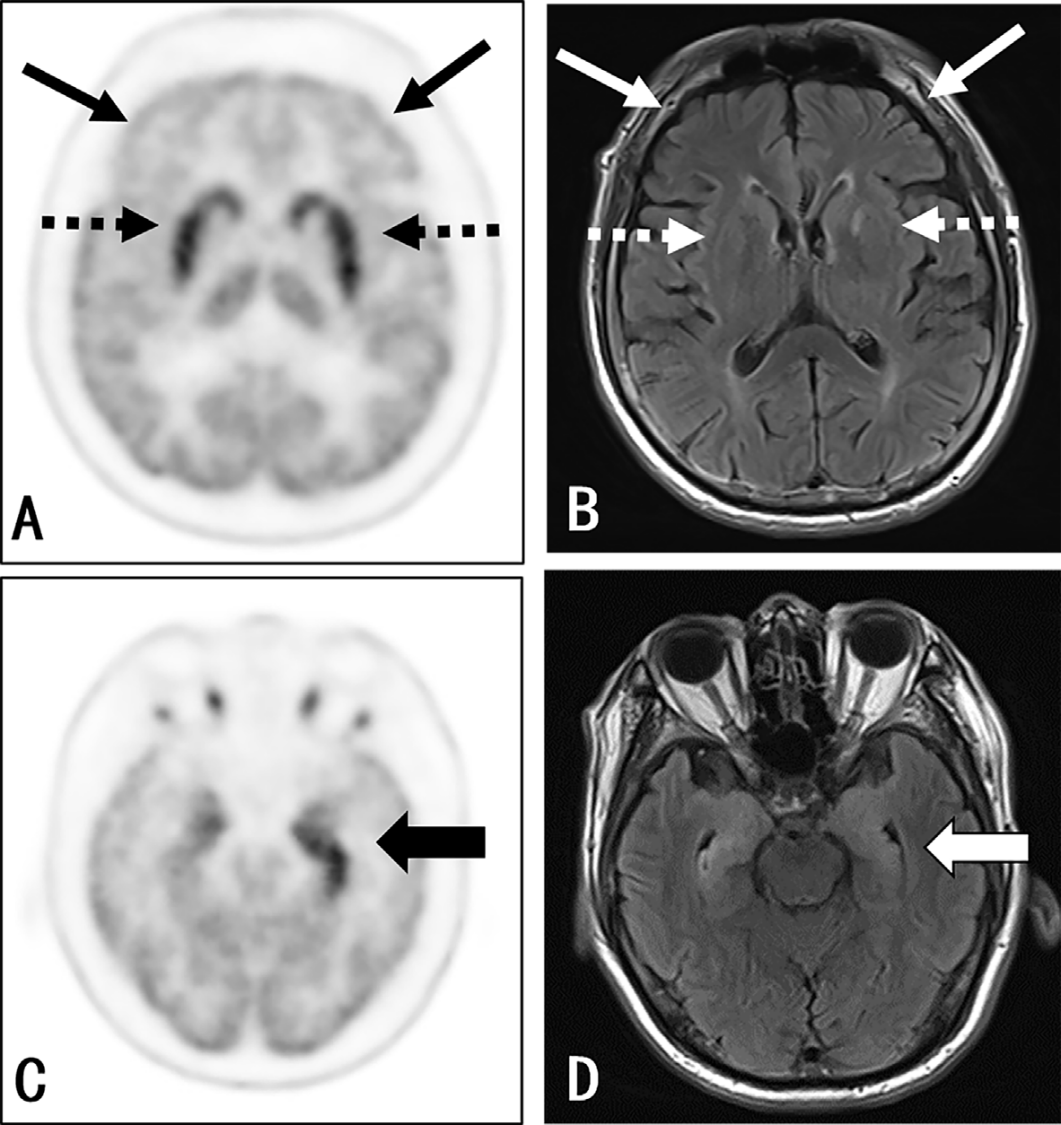
Participant 22: A 67-year-old man with a 1-month history of epileptic seizures. Positive anti-LGI1 receptor antibodies were detected in both the serum and CSF samples. The axial 3D 18F-FDG PET image at the level of the basal ganglia **(A)** and medial temporal lobe **(C)** reported an increased uptake at the medial temporal lobe (thick black arrow) (left side > the right side) and bilateral striatum (black dash arrow), along with a decreased uptake in almost all neocortex, especially in the bilateral frontal lobe (thin black arrow). The axial T2WI/FLAIR MRI **(B, D)** shows an abnormal signal in the bilateral medial temporal (right side > left side) (thick white arrow). There was no evidence of abnormality in the basal ganglia (white dash arrow) and frontal cortex (thin white arrow) as well as other cortical region, except for the nonspecific change at the left-hind limb of the internal capsule.

**Table 2** presents the comparison of the visual analysis of 18F-FDG PET scans with the MRI scans. On visual inspection, 19 (76%) patients presented with abnormal metabolism on the 18F-FDG PET. The brain regions that revealed the hypermetabolism included the hippocampus (18 [95%]), putamen (15 [79%]) and caudate (13 [68%]). The brain regions that revealed the hypometabolism included the frontal lobe (11 [58%]), parietal lobe (8 [42%]), occipital lobe (2 [11%]), and temporal lobe (1 [5%]) (**Figure 1**). Considering the metabolic pattern of visual evaluation may be influenced by the disease course (24, 25), the images findings of patients with PET scans < 12 weeks from symptom onset and those with PET scan ≥ 12 weeks from symptom onset were further analyzed. According to the diagnostic guideline of AE (1) and other studies on anti-NMDA encephalitis (8, 26, 27) and Hashimoto’s encephalopathy (28), twelve weeks (approximately 3 months) was selected as the recommended time point to divide the acute/subacute and chronic/recovery stage. There were 15 patients whose duration time from symptom onset to PET scans was less than 12 weeks. A total of 10 patients had more than 12 weeks between symptom onset and PET scans. There were 11 (73%) patients with hippocampal hypermetabolism on either side and 9 (60%) patients with putamen hypermetabolism on either side in less than 12 weeks group. Further, there were 7 patients (70%) with increased hippocampus on either side and 6 patients (60%) with increased putamen metabolism on either side in more than 12 weeks group. There was no significant difference in the proportion of hypermetabolism in hippocampus and putamen between these two groups (**Supplemental Table 2**).

**Table 2:** Imaging findings from visual inspection of the patients included in the study.

Patient no.	18F-FDG PET results			Brain T2/FLAIR MRI interpretation	Regions with increased signal in T2/FLAIR MRI
	Visual PET interpretation	Regions with significant metabolic changes by visual inspection			
		Hypermetabolism	Hypometabolism		
1	Abnormal	L CAU; L PUT; B HIP	B FRO; B PAR	Abnormal	B medial TMP
2	Abnormal	B HIP	R PAR	Abnormal	L medial TMP
3	Abnormal	B HIP	_	Abnormal	B medial TMP
4	Abnormal	B PUT; B HIP	_	Abnormal	B medial TMP
5	Abnormal	B PUT; B HIP	R FRO; R OCC	Abnormal	B medial TMP
6	Abnormal	B CAU; B PUT	B FRO; B PAR	Abnormal	B medial TMP
7	Abnormal	R CAU; B PUT; B HIP	_	Normal	_
8	Abnormal	B CAU; B PUT; B HIP	B FRO; R TMP	Normal	_
9	Normal	_	_	Normal	_
10	Abnormal	B CAU; B PUT; B HIP	B FRO; B PAR	Abnormal	B CAU; B PUT
11	Abnormal	B HIP	R FRO; R PAR	Abnormal	R medial TMP
12	Abnormal	B CAU; B PUT; B HIP	B OCC	Abnormal	R medial TMP
13	Abnormal	B CAU; B PUT; B HIP	B FRO	Abnormal	B medial TMP
14	Normal	_	_	Normal	_
15	Normal	_	_	Normal	_
16	Abnormal	B CAU; B PUT; B HIP	_	Abnormal	L medial TMP
17	Abnormal	B CAU; B PUT; B HIP	B FRO	Normal	_
18	Abnormal	B CAU; B PUT; B HIP	B FRO	Abnormal	B medial TMP
19	Abnormal	B CAU; B PUT; B HIP	_	Abnormal	B medial TMP
20	Normal	_	_	Normal	_
21	Normal	_	_	Normal	_
22	Abnormal	B CAU; B PUT; B HIP	B FRO; B PAR	Abnormal	B medial TMP
23	Abnormal	B CAU; B PUT; B HIP	B FRO; R PAR	Normal	_
24	Normal	_	_	Normal	_
25	Abnormal	B HIP	B PAR	Normal	_

B, bilateral; L, left; R, right; CAU, caudate; FRO, frontal lobe; PAR, parietal lobe; TMP, temporal lobe; OCC, occipital lobe; HIP, hippocampus; PUT, putamen.

Positive MRI findings suggestive of encephalitis were observed in 14 (56%) patients. The most common MRI findings in these patients was increased T2/FLAIR signals in the medial temporal lobes (13, 93%), which included the bilateral medial temporal lobes (9, 64%), left medial temporal lobe (2, 14%), and right medial temporal lobe (2, 14%). Merely one (7%) patient presented with basal ganglia MRI signal abnormalities.

### Imaging Findings From the Voxel Analysis

#### The Hippocampus and Cortex

**Figure 2:**
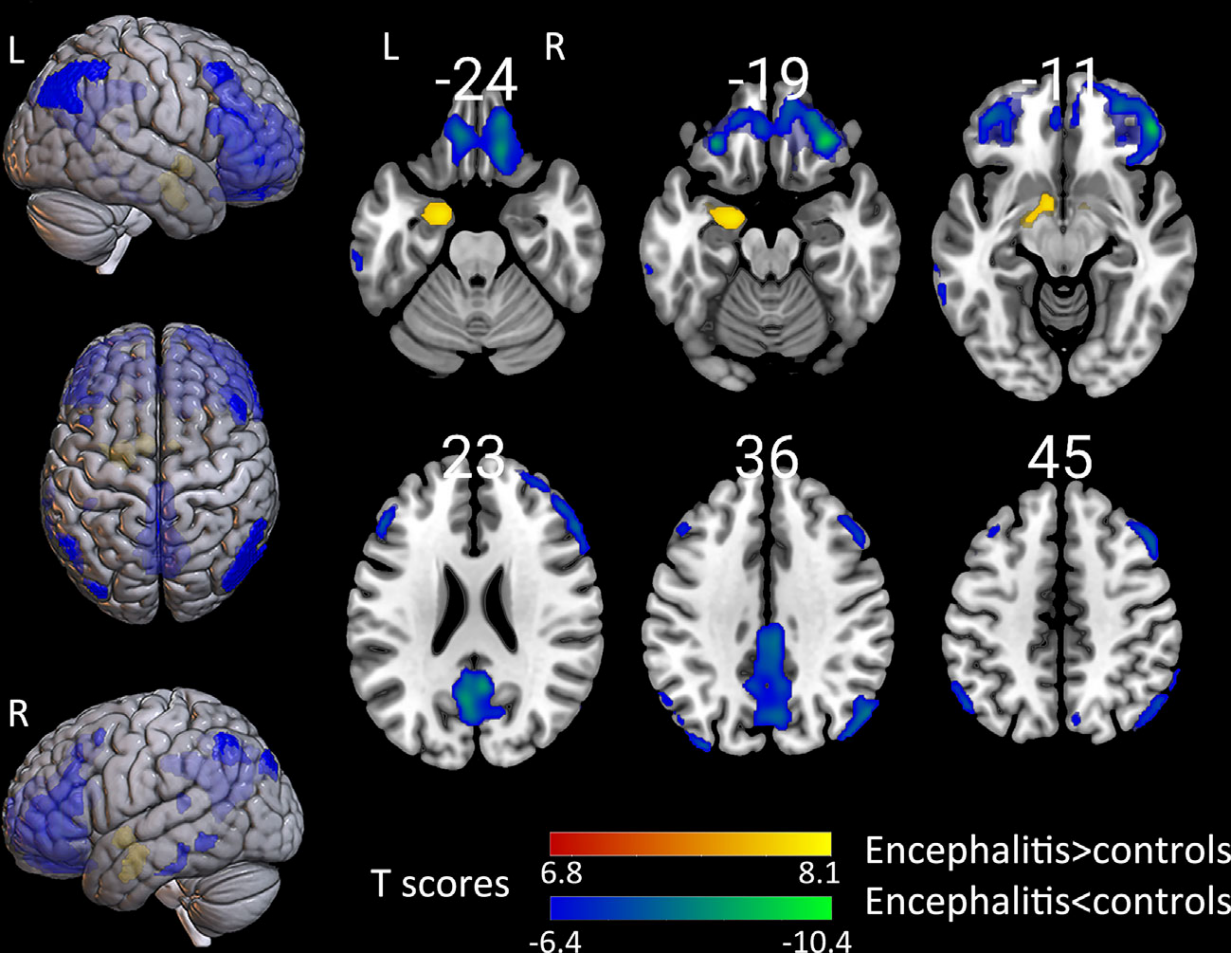
The comparison of metabolism using the 18F-FDG PET scans of patients with encephalitis and healthy controls by SPM analysis. The T-maps show the hypo-metabolism (cold) and hyper-metabolism (hot colors). The glucose metabolism increased in the left hippocampal region, and decreased in the extensive bilateral prefrontal cortex, bilateral parietal, and limited left temporal cortex. The SPM T-maps were projected onto surface, rendering and axial views of an MNI 152 template. The number in each transaxial brain image indicates the distance (mm) from the anterior commissure-posterior commissure plane. P = 0.001 was corrected. R, right; L, left.

The groupwise revealed a significantly increased uptake in the left hippocampus, para hippocampus, and amygdala. Patients presented with a decreased standard uptake in almost all neocortical regions. Particularly, hypometabolism extend in the frontal lobe was larger than in other brain areas, including the superior, middle, and inferior frontal gyrus, as well as the orbito-frontal lobe (**Figure 2**). Furthermore, hypometabolism was observed in the parietal lobe, including the left inferior parietal gyrus, bilateral precuneus, left calcarine, and right angular gyrus. Moreover, hypometabolism was observed in the left middle occipital gyrus, as well as in the left superior, middle, and inferior temporal gyrus. The individual-level results varied among patients, with most patients presenting a decreased metabolism in the frontal, parietal, occipital, and temporal lobe, as well as an increase in metabolism in the hippocampus and basal ganglia. However, some patients presented with an increase in metabolism in the neocortical region (**Supplemental Figure 1**).

The metabolic abnormality revealed by the SPM analysis and visual inspection was indicative of the hypermetabolism in the hippocampus, and basal ganglia associated with neocortical hypometabolism. With caution, these aforementioned observations can be interpreted to result from similar mechanisms. The investigators calculated the individual-level SUVr ratio for the hyper- to hypo-metabolism brain regions based on the relative 18F-FDG uptake in the anatomically defined hippocampus, putamen, and neocortical regions. Then, the investigators analyzed the relationship of the SUVr ratio of the hypermetabolic region (hippocampus and putamen) to hypometabolic region (all neocortical regions) with ADLs. The SUVr ratio of the hippocampus to neocortical region was positively associated with the ADLs (frontal lobe, r=0.529, P=0.008; parietal lobe, r=0.474, P=0.019; occipital lobe, r=0.413, P=0.045; temporal lobe, r=0.490, P=0.015), respectively. The SUVr ratio of the putamen to neocortical region was not correlated with the ADLs. Previous findings (16) and the present group-analysis results suggest that anti-LGI1 antibodies tend to affect the unilateral brain. Therefore, the investigators analyzed the association of metabolic changes in each hemisphere with ADLs. The SUVr ratio of the left hippocampus to the left frontal lobe was positively correlated with the ADLs (r=0.538, P<0.05). Furthermore, the SUVr ratio of the left putamen to the left frontal lobe was not correlated with the ADLs, which is consistent with the results for the right sides (P>0.05) (**Table 3**).

**Table 3:** Ratio of hippocampus to cortex correlation with scaled activities of daily living.

Ratio	*r*	*p*
Hippocampus/Frontal lobe	0.529	0.008
Hippocampus/Parietal lobe	0.474	0.019
Hippocampus/Occipital lobe	0.413	0.045
Hippocampus/Temporal lobe	0.490	0.015
L_Hippocampus/L_Frontal lobe	0.538	0.007
L_Hippocampus/L_Parietal lobe	0.488	0.015
L_Hippocampus/L_Occipital lobe	0.457	0.025
L_Hippocampus/L_Temporal lobe	0.534	0.007

L, left.

#### Basal Ganglia

**Figure 3:**
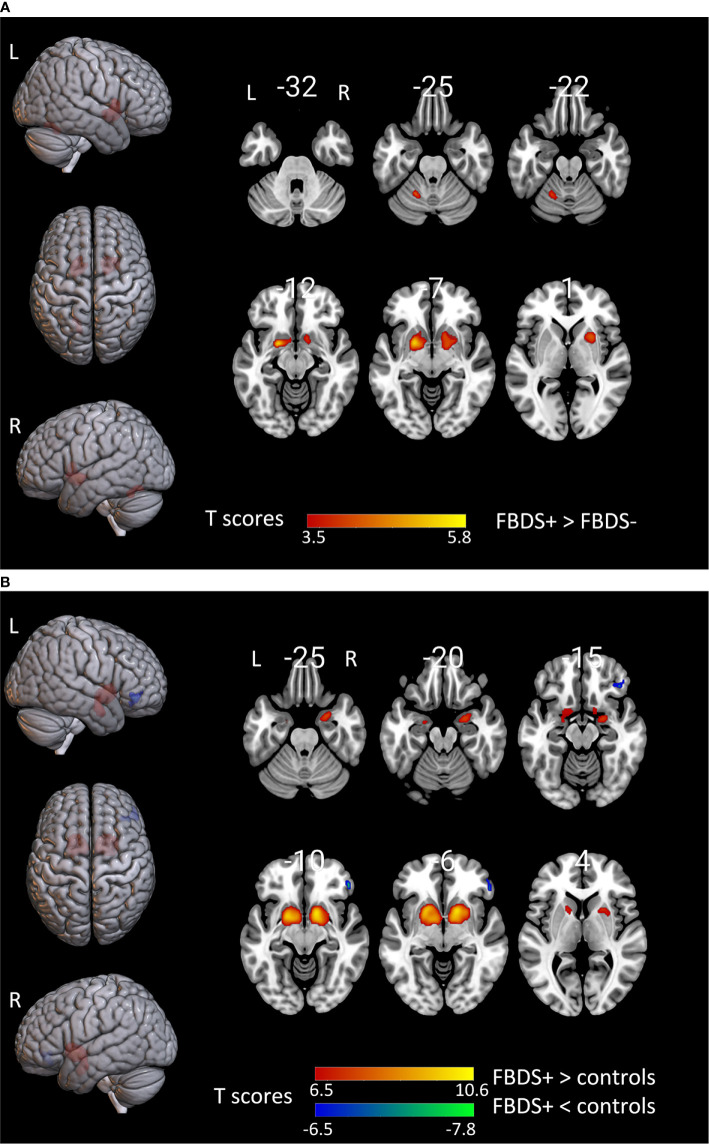
The comparison of metabolism using the 18F-FDG PET scans of patients with FBDS and those without FBDS and healthy controls by SPM analysis. The T-maps show the hypo-metabolism (cold) and hyper-metabolism (hot colors). The SPM T-maps were projected onto surface, rendering and axial views of the MNI 152 template. The number in each transaxial brain image indicates the distance (mm) from the anterior commissure-posterior commissure plane. **(A)** Compared to patients without FBDS, the glucose metabolism increased in the bilateral putamen and left cerebellum of patients with FBDS. P = 0.001 without correction. R, right; L, left.**(B)** Compared to healthy controls, the glucose metabolism increased in the bilateral putamen, and decreased in the right frontal lobe of patients with FBDS. P = 0.001 was corrected. R, right; L, left.

Previous studies have shown that 18F-FDG hyperactivity in the basal ganglia of patients may be correlated to FBDS development (2, 29). In order to explore the metabolic pattern of patients with FBDS, the metabolic pattern of patients with FBDS was compared with that of patients without FBDS and healthy controls, respectively. The groupwise revealed a significant increase uptake in the bilateral putamen in patients with FBDS, when compared to those without FBDS (**Figure 3A**). Furthermore, left cerebellum hypermetabolism was also observed. In addition, bilateral putamen hypermetabolism was found in the FBDS group, when compared to healthy controls (**Figure 3B**). Moreover, right focal frontal lobe hypometabolism was found in patients with FBDS. The metabolic difference between those without FBDS and healthy controls was similar to the difference between all included patients and healthy controls.

Similar to visual inspection, the images findings of patients with PET scans < 12 weeks from symptom onset and those with PET scan ≥ 12 weeks from symptom onset were evaluated from the voxel analysis. Voxel analysis at groupwise level showed there was no significant cluster between these two groups. Further, for voxel-based analysis at individual level, there were 10 (66.7%) patients with hippocampal hypermetabolism on either side and 8 (53.3%) patients with putamen hypermetabolism on either side in less than 12 weeks group. Further, there were 8 patients (80%) with increased hippocampus on either side and 4 patients (40%) with increased putamen metabolism on either side in more than 12 weeks group. There was no significant difference in the proportion of hypermetabolism in hippocampus and putamen between these two groups (**Supplemental Table 2**).

## Discussion

The present study revealed that the topography of anti-LGI1 encephalitis characterized by the hypermetabolism of the hippocampal and basal ganglia associated with the hypometabolism of the cortical areas. Furthermore, the metabolic abnormality extent between the hippocampus and the cortical areas was correlated with the neurological disability of these patients.

Some previous studies have focused on exploring the topography of the subtypes of AE, in order to provide characteristic metabolic patterns for the differential diagnosis (17, 30, 31). Among these, merely one study investigated the topography of certain anti-LGI1 encephalitis. This 18F-FDG PET study focused on four patients with anti-LGI1 encephalitis through SPM analysis, and discovered the topography of anti-LGI1 encephalitis was partly consistent with the present study. This study revealed the hypermetabolism in the cerebellar, basal ganglia and occipital lobe, rather than in the hippocampal, as well as the hypometabolism that was limited to the anterior cingulate/frontomesial cortex (17). This discrepancy with the present findings could be partly attributable to the differences in sample size and disease course. When compared with all four patients with an mRS score of 2, which indicated a relatively mild disease status, these present patients had a moderate status with an ADLs of 2.

The clinicopathological basis of this metabolic characteristic of anti-LGI1 encephalitis AE remains unclear. A previous study conducted a pathological analysis of patients with anti−LGI1 encephalitis, and reported the T−cell infiltration in the hippocampus and amygdala (32). Direct T−cell-mediated toxicity could result in increased ^18^F-FDG uptake in the corresponding brain region (33, 34). Another previous study reported LGI1 antibodies expression in the hippocampus and neocortex (35). This could partly explain the abnormal metabolic pattern in the neocortical region, as well as in the hippocampal region. Furthermore, patients with anti-LGI1 encephalitis presented with large-scale functional network disruptions, in addition to the hippocampal damage, which contributed to the extra-limbic clinical manifestations (36, 37). Given the higher gradient of metabolic abnormalities in the hippocampus and neocortex in patients with clinical neurologic disability, it was speculated that a higher gradient is indicative of more severe inflammation and functional connectivity impairment in the brain. However, there is a need for longitudinal studies based on the analysis of brain metabolic functional connectivity.

The SPM group-wise analysis revealed the increase in left hippocampal glucose metabolism. Furthermore, the extent of the left hippocampus hypermetabolism and cortical hypometabolism was correlated with the neurological disability. The common left hippocampal abnormalities in patients with anti-LGI1 encephalitis could be partially due to the asymmetric distribution of LGI1 expression in the human brain. A study on 36 tissue sections of the hippocampus and globus pallidus obtained from humans reported the LGI1 expression was mainly in the left hemisphere (16). Similarly, a study reported that the LGI1-antibody encephalitis affected asymmetrically only one hemisphere, which was contralateral to the affected limb for several months (18).

The hypermetabolism in the striatum could be a useful biomarker for early-stages encephalitis, before apparent EEG, MRI, or CSF abnormalities can be observed in patients with FBDS (2, 14, 29). Partially consistent with the present study, in a previous study conducted on five patients with FBDS, the 18F-FDG PET study revealed the hypermetabolism in the not only the striatum, bilaterally, and the amygdala, but also in the motor primary cortex, as well as the hypometabolism in the prefrontal cortex and right parietal associative areas (18). This discrepancy may arise from the different threshold condition for the image analysis. Compared with the P<0.05 corrected for multiple tests using the false discovery rate method, the present study used a more stringent condition with P<0.001 and the FWE correction to compare patients with the controls.

The results of PET analysis between patients with scans < 12 weeks from symptom onset and those ≥ 12 weeks from symptom onset showed no significant difference in the present study. Although some cases have reported that the duration of symptom onset is related to the metabolic pattern of autoimmune encephalitis (11, 24, 38, 39), whether and how the duration of symptom onset influences the metabolic pattern has not been concluded in a large sample prospective study. Compared with the duration of symptom onset, daily activities score of patients can reflect objectively the stage of autoimmune encephalitis. The median ADLs in this study was 2 (interquartile range: 1.5), indicating that most patients were in acute or subacute stage.

The present study has several limitations. First, this was a retrospective study that included patients from a single tertiary medical center, which could have resulted in selection bias. Second, the investigators normalized brain activity values to the average cerebellar activity rather than the average whole-brain activity, and further studies are required to verify the current findings. Third, eight patients with anti-LGI1 encephalitis in the present study were treated with steroids within 24 hours of the initial brain 18F-FDG PET. Since the corticosteroid effect could decrease the generalized cortical metabolism (40), it should be carefully noted that the effect on the whole-brain gradient metabolism pattern was slight. Finally, the antibodies were tested by fixed CBAs. Compared to a live CBAs, the fixed LGI1antibody CBAs can fail to detect some patients with low levels of LGI1 antibodies (41). Considering the present study was retrospective in nature, this factor has little impact on the results.

Overall, it was observed that subcortical hypermetabolism associated with cortical hypometabolism was the generalized topography pattern of this syndrome. Furthermore, the extent of this metabolic change between hippocampus and neocortical region was correlated with the clinical neurologic disability. When considering anti-LGI1 encephalitis has many phenotypes, including FBDS, grand mal seizures, focal aware motor seizures (FAMS), focal impaired awareness (FIAS) as well as hyponatremia. Further FAMS have different metabolic pattern from FBDS and FIAS (42) and hyponatremia may contribute to aberrant brain metabolism (43), there is a need for further prospective study to clarify the impacts of different phenotype on the metabolic characteristics of anti-LGI1encephalitis.

## Conclusions

In conclusion, hippocampal and basal ganglia hypermetabolism co-existing with neocortical hypometabolism is a common metabolic abnormality in anti-LGI1 encephalitis. Furthermore, the extent of the metabolic gradient between the hippocampus and neocortical regions was positively correlated with the neurological disability. In addition, basal ganglia hypermetabolism may contribute to the development of FBDS.

## Data Availability Statement

The raw data supporting the conclusions of this article will be made available by the authors, without undue reservation.

## Ethics Statement

Ethical review and approval was not required for the study on human participants in accordance with the local legislation and institutional requirements. Written informed consent to participate in this study was provided by the participants’ legal guardian/next of kin.

## Author Contributions

XZ: analysis and interpretation of data, and drafting manuscript. SZ: analysis and interpretation of data, and revising the manuscript. YC and ZZ: interpretation of data and revising the manuscript. XTL: analysis and interpretation of data. XL and RL: interpretation of data. QW and LA: design and conceptualization of the study, analysis and interpretation of data, and revising the manuscript. All authors contributed to the article and approved the submitted version.

## Funding

This work was supported by funds from the National Natural Science Foundation of China (81527805), the National Natural Science Foundation of China (2018YFC1315201), and Beijing Natural Science Foundation (81771143).

## Conflict of Interest

The authors declare that the research was conducted in the absence of any commercial or financial relationships that could be construed as a potential conflict of interest.

## Publisher’s Note

All claims expressed in this article are solely those of the authors and do not necessarily represent those of their affiliated organizations, or those of the publisher, the editors and the reviewers. Any product that may be evaluated in this article, or claim that may be made by its manufacturer, is not guaranteed or endorsed by the publisher.
