# Supplementary material for: Subcortical Hypermetabolism Associated With Cortical Hypometabolism Is a Common Metabolic Pattern in Patients With Anti-Leucine-Rich Glioma-Inactivated 1 Antibody Encephalitis
Source: Front Immunol. 2021 Sep 20;12:672846. doi: 10.3389/fimmu.2021.672846 (PMC8488294; doi:10.3389/fimmu.2021.672846)
Supplement: Supplementary file 1 [file DataSheet_1.docx]

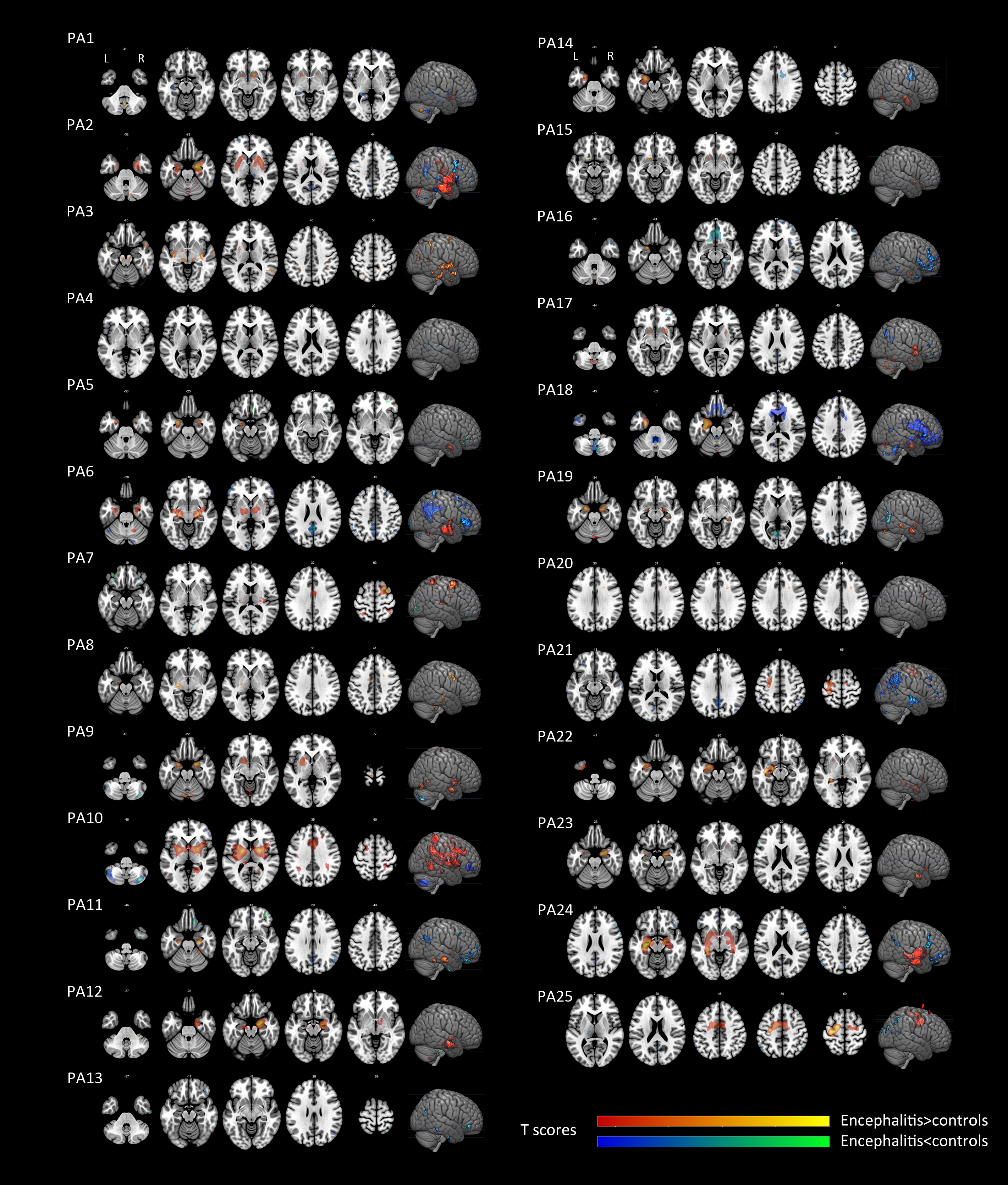


**Figure 1.** Individual-level SPM analysis of regions in patients with encephalitis compared with healthy controls. T-maps show hypo- (cold) and hyper-metabolism (hot colors). Individual analysis varied across the patients. Glucose metabolism was increased in the hippocampus and basal ganglia and decreased in the frontal, parietal, occipital, and temporal cortical regions in most patients. Some patients showed increased metabolism in the neocortical region and metabolic abnormalities in the cerebellum. SPM T-maps are projected onto a surface rendering and axial views of the MNI 152 template. All P = 0.001, uncorrected. PA= patient; R = right; L = left.

**Table 1.** The summarized brain interest regions obtained from the brain region of the AAL template.

| No. | Brain region of AAL template | Summarized brain region |
| --- | --- | --- |
| 1 | Precentral_L | Frontal Lobe |
| 2 | Precentral_R | Frontal Lobe |
| 3 | Frontal_Sup_L | Frontal Lobe |
| 4 | Frontal_Sup_R | Frontal Lobe |
| 5 | Frontal_Sup_Orb_L | Frontal Lobe |
| 6 | Frontal_Sup_Orb_R | Frontal Lobe |
| 7 | Frontal_Mid_L | Frontal Lobe |
| 8 | Frontal_Mid_R | Frontal Lobe |
| 9 | Frontal_Mid_Orb_L | Frontal Lobe |
| 10 | Frontal_Mid_Orb_R | Frontal Lobe |
| 11 | Frontal_Inf_Oper_L | Frontal Lobe |
| 12 | Frontal_Inf_Oper_R | Frontal Lobe |
| 13 | Frontal_Inf_Tri_L | Frontal Lobe |
| 14 | Frontal_Inf_Tri_R | Frontal Lobe |
| 15 | Frontal_Inf_Orb_L | Frontal Lobe |
| 16 | Frontal_Inf_Orb_R | Frontal Lobe |
| 17 | Rolandic_Oper_L | Frontal Lobe |
| 18 | Rolandic_Oper_R | Frontal Lobe |
| 19 | Supp_Motor_Area_L | Frontal Lobe |
| 20 | Supp_Motor_Area_R | Frontal Lobe |
| 21 | Olfactory_L | Frontal Lobe |
| 22 | Olfactory_R | Frontal Lobe |
| 23 | Frontal_Sup_Medial_L | Frontal Lobe |
| 24 | Frontal_Sup_Medial_R | Frontal Lobe |
| 25 | Frontal_Med_Orb_L | Frontal Lobe |
| 26 | Frontal_Med_Orb_R | Frontal Lobe |
| 27 | Rectus_L | Frontal Lobe |
| 28 | Rectus_R | Frontal Lobe |
| 29 | Hippocampal_L | Hippocampal |
| 30 | Hippocampal_R | Hippocampal |
| 31 | Putamen_L | Putamen |
| 32 | Putamen_R | Putamen |

**Table 2.** Analysis of patients with different duration time from symptom onset to PET scans

|  | <12w  (n=15) | ≥12w  (n=10) | χ^2^ | P |
| --- | --- | --- | --- | --- |
| Visual inspection |  |  |  |  |
| *Hypermetabolism in hippocampus* *n (%)* | 11(73%) | 7(70%) | 0.33 | 0.601 |
| *Hypermetabolism in putamen n (%)* | 9(60%) | 6(60%) | <0.01 | 0.659 |
| Voxel based analysis |  |  |  |  |
| *Hypermetabolism in hippocampus n (%)* | 10(66.7%) | 8(80%) | 0.53 | <0.467 |
| *Hypermetabolism in putamen n (%)* | 8(53.3%) | 4(40%) | 0.43 | 0.513 |
